# First experiences in using a dose control system on a TomoTherapy Hi·Art II

**DOI:** 10.1120/jacmp.v16i3.5489

**Published:** 2015-05-08

**Authors:** Zoë R. Moutrie, Craig M. Lancaster, Litang Yu

**Affiliations:** ^1^ Cancer Care Services Royal Brisbane and Women's Hospital Herston QLD Australia

**Keywords:** tomotherapy, dose control system, TQA, quality assurance

## Abstract

The purpose of this study was to investigate the impact of a dose control system (DCS) servo installed on two fully commissioned TomoTherapy Hi·Art II treatment units. This servo is designed to actively adjust machine parameters to control the output variation of a tomotherapy unit to within ±0.5% of the nominal dose rate. Machine output, dose rate, and patient‐specific quality assurance data were retrospectively analyzed for periods prior to and following the installation of the servo system. Quality assurance tests indicate a reduction in the rotational variation of the output during a procedure, where the peak‐to‐peak amplitude of the variation was ±1.30 prior to DCS and equal to ±0.4 with DCS. Comparing two tomotherapy unit static outputs over four years the percentage error was 1.05%±0.7% and −0.4%±0.66% and, once DCS was installed, was reduced to −0.22%±0.29% and −0.08%±0.16% . The results of the quality assurance tests indicate that the dose control system reduced the output variation of each machine for both static and rotational delivery, leading to an improvement in the overall performance of the machine and providing greater certainty in treatment delivery.

PACS number: 87.56.‐v, 87.56.bd, 87.55.Qr, 87.56.Fc

## I. INTRODUCTION

Helical tomotherapy is a treatment modality providing an intensity‐modulated delivery of megavoltage X‐rays, synchronizing the rotation of the radiation beam with the patient couch which translates through the ring gantry, resulting in a helical intensity‐modulated arc therapy.^1^, ^2^, ^3^, ^4^, ^5^, ^6^ The treatment delivery is a function of radiation beam‐on time, and two sealed parallel plate transmission ionization chambers are used to monitor the dose rate and terminate the beam should the dose rate exceed specifications described by Langden et al.^(4)^ TomoTherapy units (Accuray Inc., Sunnyvale, CA) utilize an integrated megavoltage on‐board imaging system^7^, ^8^, ^9^ for image‐guided treatment deliveries^6^, ^10^ and, since the source of the imaging beam and the treatment beam are the same, users can be assured of no misalignment due to mechanical shifts between the imaging and treatment isocenters^11^, ^12^ This MVCT system consists of a 640 channel Hitachi CT (Hitachi, Ltd., Tokyo, Japan) system which utilizes 520 channels for imaging and up to 575 channels for other procedures. The MVCT system is employed in the commissioning of the tomotherapy units for a variety of tests^4^, ^13^, ^14^ and routine QA,^(15)^ and has been shown to be of use in patient dosimetric and positioning verification^(16)^ and computation of patient doses.^(17)^

The treatment planning system (Accuray Inc.) used at the Royal Brisbane and Women's Hospital (RBWH) assumes a constant output and constant size and shape of the longitudinal and transverse profiles, and the dose to each patient voxel is the summation of multiple beamlets.

Tomotherapy units have been found to suffer from output variation^18^, ^19^ with gantry angle^(20)^ and also with time, and it has been suggested that, if the output remains within 2% of the average, this effect will not produce clinically significant variations from planned doses.^4^, ^21^ This output variation has been shown in some cases to have a minimal clinical impact^19^, ^22^, ^23^ since each planned dose voxel is typically irradiated by multiple beamlets (over multiple gantry angles). However, at the RBWH, treatment delivery quality assurance failures are still often attributed to this variation and to ameliorate these effects, Accuray Inc. introduced the Dose Control System (DCS) which uses feedback from the monitor chambers in a closed‐loop feedback system to stabilize the beam output. This system is implemented to control the output variation of a tomotherapy unit to within ±0.5% of the nominal dose rate by adjusting the pulse amplitude control and the injector current.

The output variation can be separated into two components: dose drift and rotational variation. The dose drift is a gradual decrease over time in the output (dose per unit time) of the machine, which is most significant within the first 200 s of beam‐on time which the manufacturer attributes to thermal effects, the increase of the temperature of components leading to a reduction in output. Some of the factors that these thermal effects are attributed to include: the differential expansion between the cathode and grid, heating of the gun caused by the radio frequency driving system, and the initial cathode temperature. The rotational variation of the output can be described as a sinusoidal change in the output as the gantry rotates; the oscillation frequency of the change corresponds to the gantry period and is caused by changes in the mechanical forces applied to the magnetron. These mechanical forces are the change in the cathode position relative to the earth's gravity and magnetism force during gantry rotation.

The purpose of this study was to evaluate the effectiveness of the DCS on two previously commissioned Dragon (Chengdu Twin Peak Accelerator Technology Inc., Sichuan, China) fixed‐target TomoTherapy units installed at the RBWH.

## II. MATERIALS AND METHODS

In this study two important aspects of quality assurance (QA) were considered — the effect of DCS on machine output parameters, and its effect on patient‐specific dose measurements. Data acquired prior to and following the installation of DCS on both tomotherapy units were evaluated. The results of the quality assurance tests prior to and following the installation of DCS on both of RBWH's tomotherapy units were assessed.

### A. TQA

At the RBWH, routine QA of the tomotherapy units includes monitoring a number of parameters acquired by the on‐board monitoring systems of the accelerator,^(21)^ and the software TomoTherapy Quality Assurance (TQA) (Accuray Inc.) described by Choi et al.^(24)^ and Coevoet et al.^(25)^ is used to report these data. Several procedures are used in this study and the results of the machine output of three of these procedures were evaluated.

#### A.1 Step‐wedge helical

This procedure utilizes data collected over 10 gantry rotations which are acquired over a 200 s period, with the jaws maintained at a fixed aperture of 5 cm. An aluminum step phantom is translated through the beam as the couch translates at a fixed speed of 1.5 mm/s. As each step of the phantom enters the beam, MLCs are opened and closed. The metrics for this test procedure audited in this investigation are the pulse‐by‐pulse output and the average helical output.

#### A.2 Step‐wedge static

The step phantom described in A.1 above is translated through a static beam (the gantry of 0°) at a constant rate of 1.5 mm/s, where the MLCs remain open for the duration of the 200 s procedure and the jaws kept at 5 cm separation. In this investigation, the pulse‐by‐pulse output and the average static output were audited to provide an indication of the static output drift during a procedure.

#### A.3 Basic dosimetry

During the 200 s procedure, no external attenuation of the beam is made, since neither a phantom nor the treatment couch is placed in the path of the beam. Machine data are collected over 10 gantry rotations, during which both the MLCs and the jaws are kept fully open. The pulse‐by‐pulse output and the average helical output were evaluated in this procedure.

### B. Static beam QA

The measurement of static output and beam energy with A1SL Shonka ionization chambers (Standard Imaging, Inc., Middleton, WI) in Virtual Water (Standard Imaging, Inc., Middleton, WI) is monitored routinely at RBWH. The chamber is placed 1.5 cm deep in the phantom along the beam central axis and irradiated for 1 min to quantify output. The chamber is then irradiated for 1 min at depths of 10 cm and 20 cm on the central axis to monitor energy. A reference chamber is placed adjacent to the measurement chamber to monitor any drift in the output with each irradiation.

### C. Delivery QA

At RBWH all patient plans are assessed with plan‐specific quality assurance (also known as delivery quality assurance (DQA)) where the delivery sequence is calculated on, and delivered to, a cylindrical Virtual Water phantom. A point‐dose measurement at 5 mm inferior to the center of the phantom is acquired with the aforementioned A1SL chamber and EDR2 radiographic film (Carestream Health, Rochester, NY) positioned at a coronal plane bisecting the phantom.^(4)^

## III. RESULTS

### A. TQA

#### A.1 Step‐wedge helical

**Figure 1:**
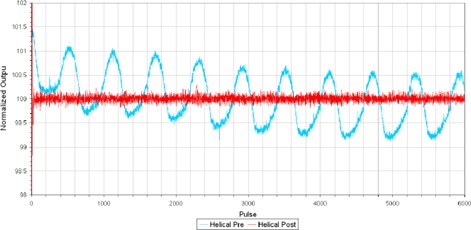
TQA normalized output variation per pulse for the helical step‐wedge procedure prior to and following the installation of DCS on one tomotherapy unit.

The rotational output change prior to and following the installation of the DCS is clearly observed in Fig. 1 where the pulse by pulse output no longer follows a sinusoidal pattern.

**Figure 2:**
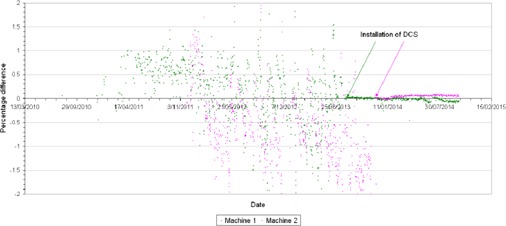
Trend of the reported average helical step‐wedge TQA results for both tomotherapy units; the arrows indicate the date of the installation of DCS.

The effect of DCS on the stability of the average output from the helical step‐wedge procedure can be clearly noted from Fig. 2, where the mean average helical step‐wedge output percentage error for each tomotherapy unit was 0.15%±0.69% and −0.67%±0.78%, and following installation was −0.01%±0.03% and 0.04%±0.04%, respectively.

#### A.2 Step‐wedge static

**Figure 3:**
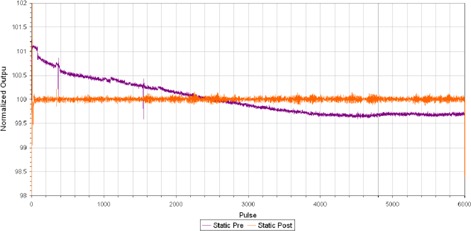
TQA normalized output variation per pulse for the static step‐wedge procedure prior to and following the installation of DCS on one tomotherapy unit.

From Fig. 3, the static step‐wedge procedure indicated a quasiexponential decrease in normalized output as a function of pulse in the absence of the DCS. However, once the DCS is installed, the output is kept constant.

The effect of DCS on the stability of the pulse‐by‐pulse output from the static step‐wedge procedure can be clearly noted from Fig. 3. In comparing the change in the average static step‐wedge output percentage for each tomotherapy unit, the average was 0.42%±0.49% and 0.37%±0.69% prior to the installation, and following was −0.02%±0.04% and 0.04%±0.03%, respectively.

#### A.3 Basic dosimetry

**Figure 4:**
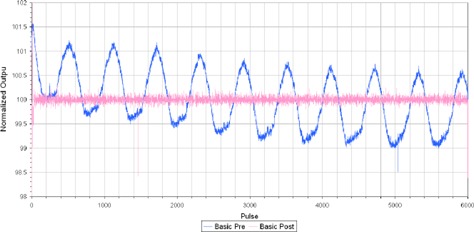
TQA normalized output variation per pulse for the basic dosimetry procedure prior to and following the installation of DCS on one tomotherapy unit.

The effect of DCS on the stability of the pulse‐by‐pulse output from the basic dosimetry procedure can be clearly noted from Fig. 4. In comparing the change in the average basic dosimetry output percentage error for each tomotherapy unit, the average was 0.08%±0.63% and 1.31%±0.92% prior to the installation, and following it was −0.03%±0.02% and 0.04%±0.49%, respectively.

### B. Static beam QA

In the absence of the DCS, each machine static output measurement percentage error was 1.05%±0.7% and −0.4%±0.66%, and with DCS was −0.22%±0.29% and −0.08%±0.16% . These static output data are now encompassed within a smaller standard deviation for both machines, indicating that the static output varies less than previously recorded.

### C. Delivery QA

The patient‐specific QA point‐dose results of both machines were assessed and the average percentage difference between measured and calculated doses for each tomotherapy unit were −0.42%±1.66% and 1.62%±1.1% prior to the DCS, and following they were 0.67%±1.14% and 0.38%±0.86%, respectively. The DQA dose measurements are found to be constrained within smaller standard deviations than prior to DCS, with most falling within 2% of planned doses.

## IV. DISCUSSION

The results of the static TQA procedures (the output as a function of pulse) in Fig. 3 demonstrate that the gradual dose drift during a procedure has been eliminated by the implementation of the dose control system. For a given 200 s helical procedure, the standard deviation of the output was ±0.66% and ±0.52% prior to DCS, and with DCS is reduced to ±0.10% and ±0.13%.

The results of the helical TQA procedures indicate a reduction in the rotational variation of the output during a procedure when DCS is installed on a TomoTherapy Hi·Art II accelerator, where the peak‐to‐peak amplitude of the variation was ±1.30% prior to DCS and equal to ±0.4% with DCS.

The interfractional (or day to day) variation of the rotational output has been shown to be reduced when comparing the average helical output results for both basic dosimetry and helical step‐wedge prior to and following the installation of a DCS on both tomotherapy units.

The patient‐specific quality assurance tests show that, on a system with DCS, a much tighter degree of agreement with the planned dose can be expected and might lead to implementation of tighter tolerances for these quality assurance tests.

The interfractional variation of the static output was observed to decrease when the average output results of the static step‐wedge procedure are compared prior to and following DCS installation. This change has been corroborated by the static ion chamber measurement of dose rate. This has resulted in less frequent adjustments of the output once each treatment unit was fitted with a DCS.

The difference between the nominal dose rate of machine 1 and machine 2 was 2.88% prior to the installation of DCS, and 0.7% following the installation. The results of all output measurements show a more consistent and stable output intra‐ and interfractionally.

## V. CONCLUSIONS

Based on the quality assurance results, the implementation of a dose control system on a TomoTherapy Hi·Art II linear accelerator has improved the inter‐ and intrafractional variation in output when compared with the performance of the same accelerators without the dose control system.

## ACKNOWLEDGMENTS

The authors acknowledge the assistance of the Mr. Jong Gi Lee for lengthy discussions on DCS operation.
